# A molecular sub-cluster of colon cancer cells with low VDR expression is sensitive to chemotherapy, BRAF inhibitors and PI3K-mTOR inhibitors treatment

**DOI:** 10.18632/aging.102349

**Published:** 2019-10-09

**Authors:** Haiwei Wang, Xinrui Wang, Liangpu Xu, Ji Zhang, Hua Cao

**Affiliations:** 1 Fujian Key Laboratory for Prenatal Diagnosis and Birth Defect, Fujian Provincial Maternity and Children’s Hospital, Affiliated Hospital of Fujian Medical University, FuZhou, FuJian 350001, China; 2 Key Laboratory of Technical Evaluation of Fertility Regulation for Non-human Primate, National Health and Family Planning Commission, FuZhou, FuJian 350001, China; 3 State Key Laboratory for Medical Genomics, Shanghai Institute of Hematology, Rui-Jin Hospital Affiliated to School of Medicine, Shanghai Jiao Tong University, Shanghai 200025, China

**Keywords:** colon cancer subtypes, chemotherapy, BRAF inhibitors, PI3K-mTOR inhibitors, VDR

## Abstract

Gene expression based consensus molecular subtypes (CMS) and non-negative matrix factorization (NMF) sub-clusters are robust colon cancer classification systems. Although, the molecular features are clear, colon cancer subgroups based interventions are limited. To address this problem, we analyze the CMS and NMF subgroup guided drug sensitivity in colon cancer cell lines. CMS3 subtype cells are sensitive to 5-Fluorouracil, while, CMS4 subtype cells are sensitive to cisplatin treatment. In NMF classification, a sub-cluster is specifically sensitive to chemotherapy, BRAF inhibitors, PI3K-mTOR inhibitors and NOTCH inhibitor treatment. This sub-cluster has low frequency of TP53, POLE, PIK3CA and BRAF mutation. Transcriptional analysis demonstrates low NOTCH signaling activity, low CDX2 and VDR expression in this sub-cluster. CDX2 and VDR are significantly associated with the sensitivity of chemotherapy, BRAF inhibitors and PI3K-mTOR inhibitors. Moreover, a positive correlation between VDR and CDX2 is identified. VDR and CDX2 mediated regulatory networks are constructed. At last, three or four sub-clusters classification is validated in colon cancer patients. Overall, our results suggest a molecular sub-cluster of colon cancer cells with low CDX2 and VDR expression is sensitive to chemotherapy, BRAF inhibitors and PI3K-mTOR inhibitors treatment and provide an example of translation of cancer classification to subgroup guided therapies.

## INTRODUCTION

Colon cancer is a heterogeneous disease with distinctive genetic and epigenetic alterations [1, 2]. The heterogeneity of colon cancer is reflected by the differences in tumor aggressiveness, pathologic features and responses to therapies [3]. There is an urgent need for robust classification of cancer subtypes to provide insight of oncogenic mechanisms and predict the therapeutic responses [4, 5].

To date, several colon cancer classification systems based on genomic alterations, gene expression profiles, DNA methylation aberrations or proteomic characteristics have been reported [6–11]. Particularly, in 2015, Justin Guinney and colleagues integrated the expression data of 4,151 patients from 18 published colon cancer datasets and proposed the CMS classification of colon cancer, including CMS1 microsatellite instability (MSI) immune, CMS2 canonical, CMS3 metabolic and CMS4 mesenchymal four classes [12]. There was prognostic significance of the CMS classification [13]. However, treatment options for each CMS sub-group patients were limited [14]. In 2013, Anjuraj Sadanandam and colleagues analyzed the expression data of 1,290 colon cancer patients from published datasets and divided those colon cancer patients into goblet-like, enterocyte, stem-like, inflammatory and transit-amplifying five subtypes based NMF classification [15]. The stem-like colon cancer was associated with the clinical benefit of FOLEIRI treatment. The transit-amplifying colon cancer was associated with the clinical benefit of EGFR inhibitor cetuximab or c-MET inhibitor treatment. However, other subgroup based targeted interventions were not further analyzed.

Moreover, the previously described colon cancer classification systems were principally focusing on the characterization of primary tumors, which contained many distinct cell types, including tumor cells, fibroblastic stroma, blood vessels and immune cells. This high level of tissue complexity could cause difficulties in interpreting the ultimate classified results across different studies [16, 17]. Alternatively, cancer cell lines are devoid of other cell types and may represent the intrinsic property of tumor. And with the available datasets in Cancer Cell Line Encyclopedia [18, 19] and Genomics of Drug Sensitivity in Cancer [20], we now could determine the biological features and potential therapeutic response of colon cancer subtypes derived from colon cancer cell lines.

So, in this study, we analyze the CMS and NMF classification systems in colon cancer cell lines and determine the subgroup specific genomic mutation and subgroup based drug response. We find that a molecular sub-cluster of colon cancer cells with low CDX2 and VDR expression is specifically sensitive to chemotherapy, BRAF inhibitors and PI3K-mTOR inhibitors treatment.

## RESULTS

### CMS3 subtype colon cancer cells are more sensitive to 5-Fluorouracil treatment and CMS4 subtype colon cancer cells are more sensitive to cisplatin treatment

**Figure 1:**
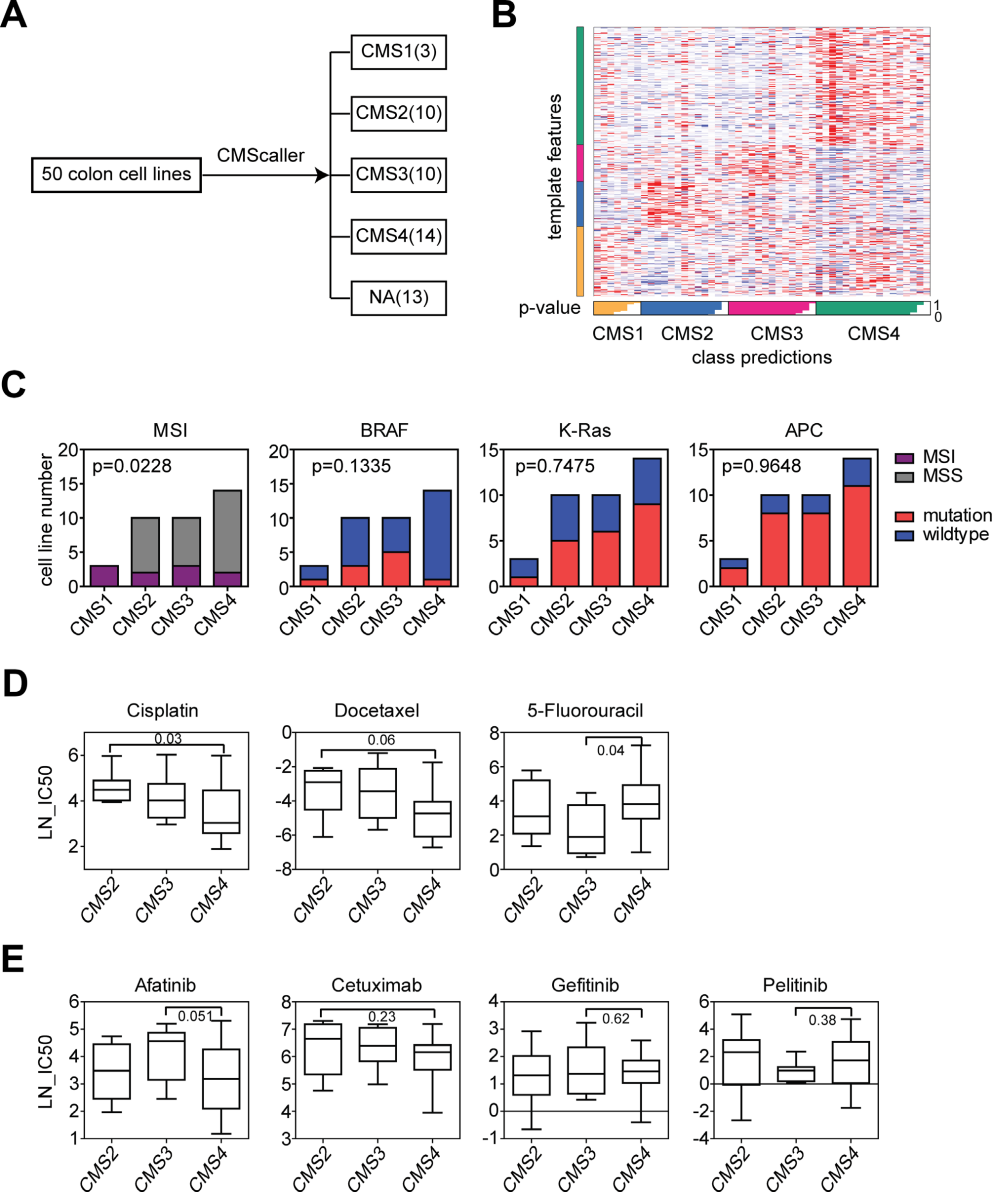
**CMS3 subtype colon cancer cells are more sensitive to 5-Fluorouracil treatment and CMS4 subtype colon cancer cells are more sensitive to cisplatin treatment.** (**A**) Colon cancer cell lines were divided into CMS subtypes based on the gene expression profiling using CMScaller. Number of colon cancer cell lines in each CMS subtype was demonstrated. (**B**) A template feature of the CMS subtypes. (**C**) Contingency graphs showed the number of MSI or MSS subtype, BRAF, K-Ras and APC mutant or wild type colon cancer cell lines in each CMS subtype. P values were determined by Chi-square test. (**D**) Box plots showed the LN-IC50 of chemotherapy drugs cisplatin, docetaxel and 5-Fluorouracil in CMS2, CMS3 and CMS4 subtypes of colon cancer cells. P values were performed using Student’s t test. (**E**) Box plots showed the LN-IC50 of EGFR inhibitors in CMS2, CMS3 and CMS4 subtypes of colon cancer cells.

We used the datasets derived from Genomics of Drug Sensitivity in Cancer project to determine the drug response in different CMS subtypes. Colon cancer cell lines were divided into CMS subtypes based on the gene expression profiling using CMScaller [21]. The number of colon cancer cell lines in each CMS subtype was demonstrated in Figure 1A. There were 13 colon cancer cell lines failed in classification into any of those four subtypes. The four CMS subtypes displayed distinctive template features (Figure 1B).

First, we determined the genomic characteristics of each CMS subtype. Cells in CMS1 subtype were all MSI (Figure 1C), consistent with the results identified in colon cancer patients [12]. However, contrast to the high frequency of BRAF mutation in CMS1 subtype and K-Ras mutation in CMS3 subtype colon cancer patients, there was no significant difference in the frequencies of BRAF and K-Ras mutation in CMS subtypes derived from colon cancer cell lines (Figure 1C). CMS2 subtype colon cancer patients were characterized by the activation of WNT signaling pathway, however, we did not observe high frequency of APC mutation in CMS2 subtype derived from colon cancer cell lines neither (Figure 1C). Those results showed some inconsistent results between colon cancer patients and colon cancer cell lines and highlighted the importance of the tumor microenvironment in determining the colon cancer subtypes.

Next, we determined the drug response of the different CMS subtypes. 5-Fluorouracil is the first-line chemotherapy regimen in colon cancer treatment [22, 23]. We found that CMS3 subtype colon cancer cells were more sensitive to 5-Fluorouracil treatment compared with CMS4 subtype (Figure 1D). CMS4 subtype colon cancer cells were more sensitive to cisplatin treatment compared with CMS2 subtype (Figure 1D). There was no significant difference in the docetaxel sensitivity in CMS subtypes (Figure 1D). The epidermal growth factor receptor (EGFR) represents an important drug target in colon cancer treatment. EGFR antibody cetuximab is wildly used for the treatment of K-Ras wild type metastatic colon cancer patients [24, 25]. However, we found that cetuximab and other EGFR inhibitors showed no drug preference in CMS subtypes (Figure 1E).

### Chemo-sensitivity is different in the three sub-clusters of colon cancer cell lines classified by NMF

**Figure 2:**
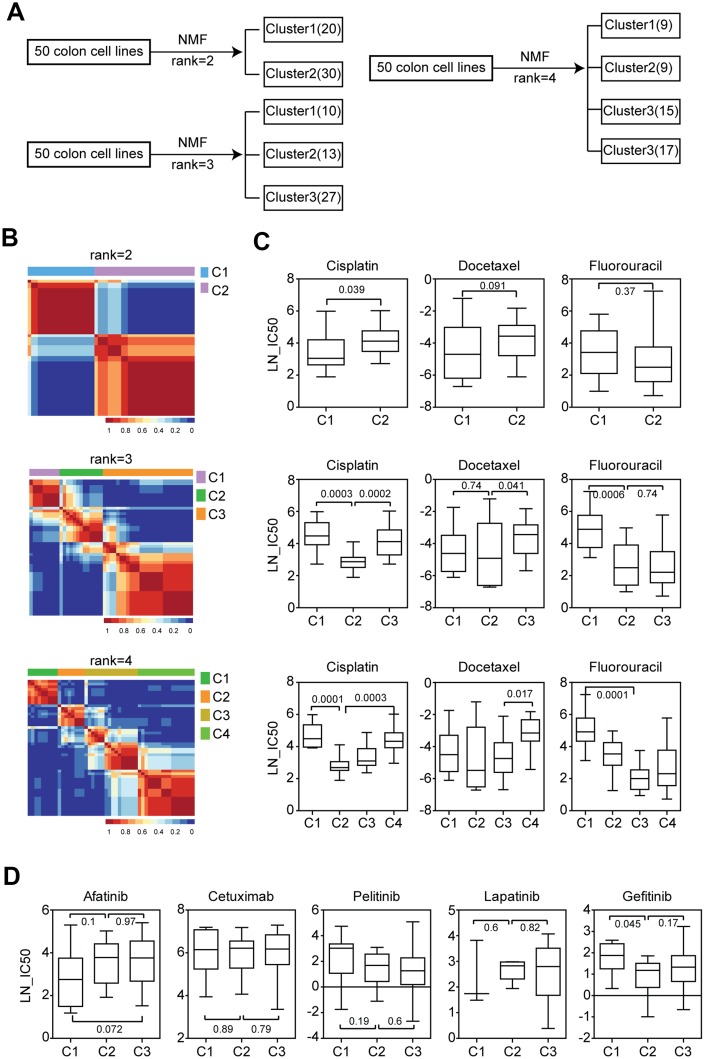
**Chemo-sensitivity is different in the three sub-clusters of colon cancer cell lines classified by NMF.** (**A**) Colon cancer cell lines were divided into two, three or four sub-clusters based on the gene expression profiling using NMF. Number of colon cancer cell lines in each sub-cluster was demonstrated. (**B**) Consensus maps showed the correlation profilings of colon cancer cell lines from two consensuses, three consensuses or four consensuses. (**C**) Box plots showed the LN-IC50 of chemotherapy drugs cisplatin, docetaxel and 5-Fluorouracil in each sub-cluster derived from two consensuses, three consensuses or four consensuses. (**D**) Box plots showed the LN-IC50 of EGFR inhibitors in each colon cancer sub-cluster derived from three consensuses.

Another important colon cancer patient classification system was using NMF [15]. Based on the NMF classification, we divided the colon cancer cell lines into two sub-clusters, three sub-clusters or four sub-clusters. The number of cell lines in each cluster was demonstrated in Figure 2A. And the consensus heatmaps were demonstrated in Figure 2B.

We tested the drug response in different colon cancer sub-clusters. When the colon cancer cell lines were divided into two sub-clusters, we found that cells in cluster1 were more sensitive to cisplatin treatment (Figure 2C). However, there was no significant difference in the docetaxel and 5-Fluorouracil sensitivity between cluster1 and cluster2 colon cancer cells (Figure 2C).

We then divided the colon cancer cell lines into three sub-clusters. Interestingly, we found that colon cancer cells in cluster2 were more sensitive to cisplatin, docetaxel and 5-Fluorouracil treatment compared with the other two clusters (Figure 2C). Particularly compared with cluster3, colon cancer cells in cluster2 were more sensitive to cisplatin and docetaxel treatment. Also compared with cluster1, colon cancer cells in cluster2 were more sensitive to cisplatin and 5-Fluorouracil treatment (Figure 2C).

Colon cancer cell lines were also divided into four sub-clusters. The results were quite similar to the findings obtained from three sub-clusters. Colon cancer cells in cluster2 and cluster3 showed higher sensitivity of cisplatin, docetaxel and 5-Fluorouracil treatment than cluster1 or cluster4 (Figure 2C). Those results provided strong supports for the presence of at least three sub-clusters of colon cancer cell lines.

We also analyzed the sensitivity of EGFR inhibitors in different sub-clusters of colon cancer cells. We found that there was no significant difference in the afatinib, cetuximab, pelitinib and lapatinib sensitivity in the colon cancer sub-clusters. Only colon cancer cells in cluster2 were more sensitive to gefitinib treatment compared with cluster1 (Figure 2D).

### Genomic differences of the three sub-clusters of colon cancer cell lines suggest the different response to BRAF inhibitors and PI3K-mTOR inhibitors treatment

**Figure 3:**
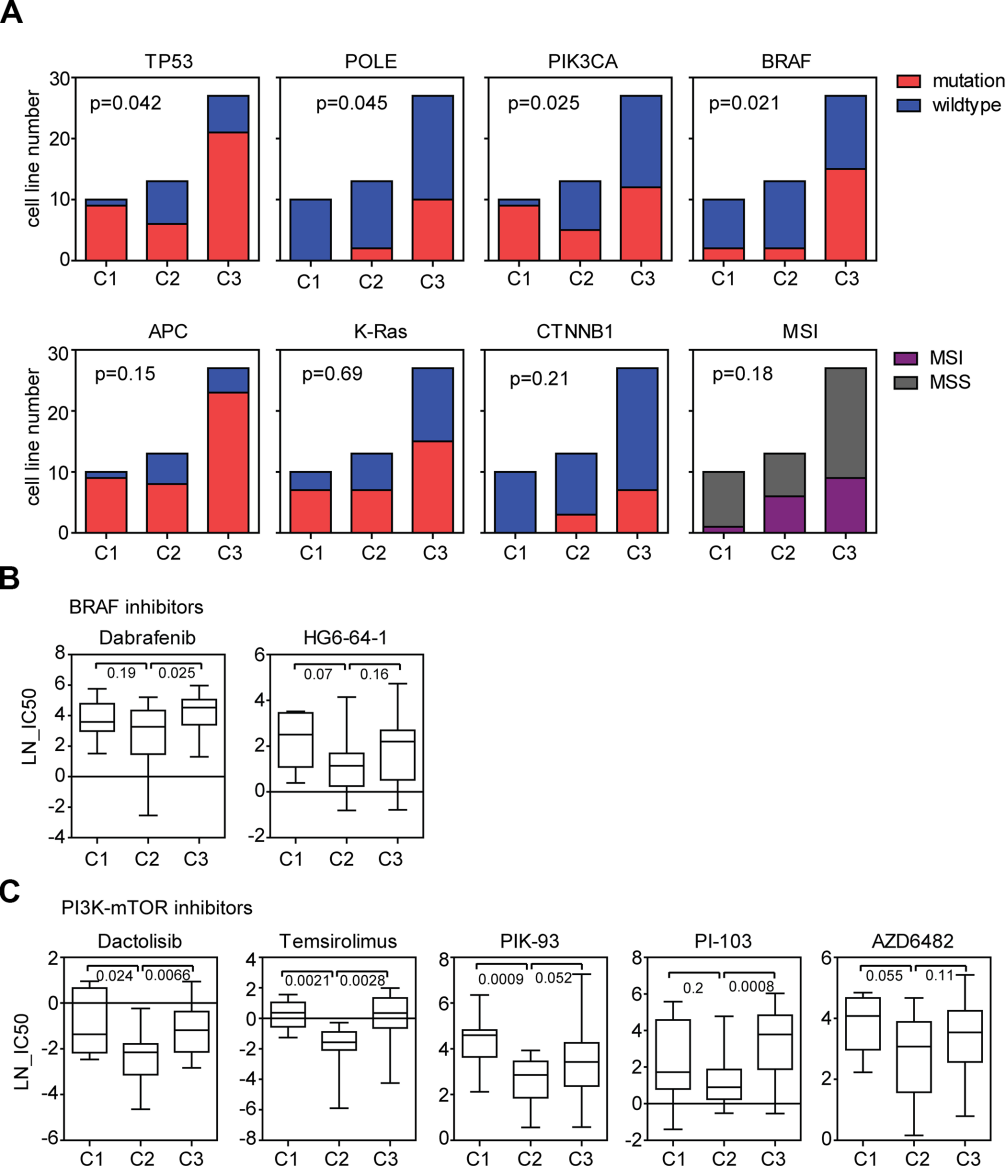
**Genomic differences of the three sub-clusters of colon cancer cell lines suggest the different response to BRAF inhibitors and PI3K-mTOR inhibitors treatment.** (**A**) Contingency graphs showed the number of TP53, POLE, PIK3CA, BRAF, APC, K-Ras and CTNNB1 mutant or wild type, MSI or MSS subtype colon cancer cell lines in each sub-cluster derived from three consensuses. P values were determined by Chi-square test. (**B**) Box plots showed the LN-IC50 of BRAF inhibitors dabrafenib and HG6-64-1 in each sub-cluster. (**C**) Box plots showed the LN-IC50 of PI3K-mTOR inhibitors in each sub-cluster.

Next, we determined the genomic characteristics of the three sub-clusters of colon cancer cells. Compared with other two clusters, colon cancer cells in cluster2 had low frequency of TP53 mutation (Figure 3A). Since TP53 was a key factor in determining the chemo-sensitivity [26], those results were consistent with the high sensitivity of cisplatin, docetaxel and 5-Fluorouracil in cluster2 colon cancer cells. Colon cancer cells in cluster2 were also strongly associated with PIK3CA, BRAF and POLE wild type status (Figure 3A). However, we did not observe different frequency of APC, K-Ras and CTNNB1 mutation in the three sub-clusters (Figure 3A). Moreover, the three sub-clusters demonstrated no difference in the MSI distribution neither (Figure 3A).

Since the three sub-clusters had different frequency of BRAF mutation (Figure 3A), we speculated that BRAF inhibitors may have different sensitivity in the three sub-clusters of colon cancer cells. Five BRAF inhibitors AZ628, HG6-64-1, PLX-4720, SB590885 and dabrafenib were analyzed. Compared with cluster3, colon cancer cells in cluster2 was more sensitive to dabrafenib treatment (Figure 3B). And less significantly, cluster2 was more sensitive to HG6-64-1 treatment compared with cluster1 (Figure 3B). However, other BRAF inhibitors showed no drug preference in the three sub-clusters.

PIK3CA is an important regulator of PI3K-mTOR signaling pathway [27]. We found that PIK3CA mutation frequency was also significantly different in the three sub-clusters of colon cancer cells (Figure 3A), so we tested the sensitivity of PI3K-mTOR signaling pathway inhibitors in different sub-clusters of colon cancer cells. Totally, eight PI3K-mTOR signaling pathway inhibitors were analyzed. We found that colon cancer cells in cluster2 were more sensitive to dactolisib, temsirolimus PIK-93 or PI-103 treatment compared with colon cancer cells in cluster1 or cluster3 (Figure 3C). And less significantly, cluster2 colon cancer cells were more sensitive to AZD6482 treatment compared with cluster1 (Figure 3C). There was no significant difference in the idelalisib, OSI-027 and pictilisib sensitivity in colon cancer sub-clusters.

With all the above results, we identified a molecular sub-cluster of colon cancer cells which was particularly sensitive to chemotherapy, BRAF inhibitors and PI3K-mTOR inhibitors treatment. However, we should emphasize that although most BRAF and PI3K-mTOR inhibitors showed preferential sensitivity to cluster2 colon cancer cells, some other BRAF and PI3K-mTOR inhibitors showed not.

### Characteristics of gene expression and functional annotation of colon cancer sub-clusters suggest the different response to NOTCH inhibitor treatment

**Figure 4:**
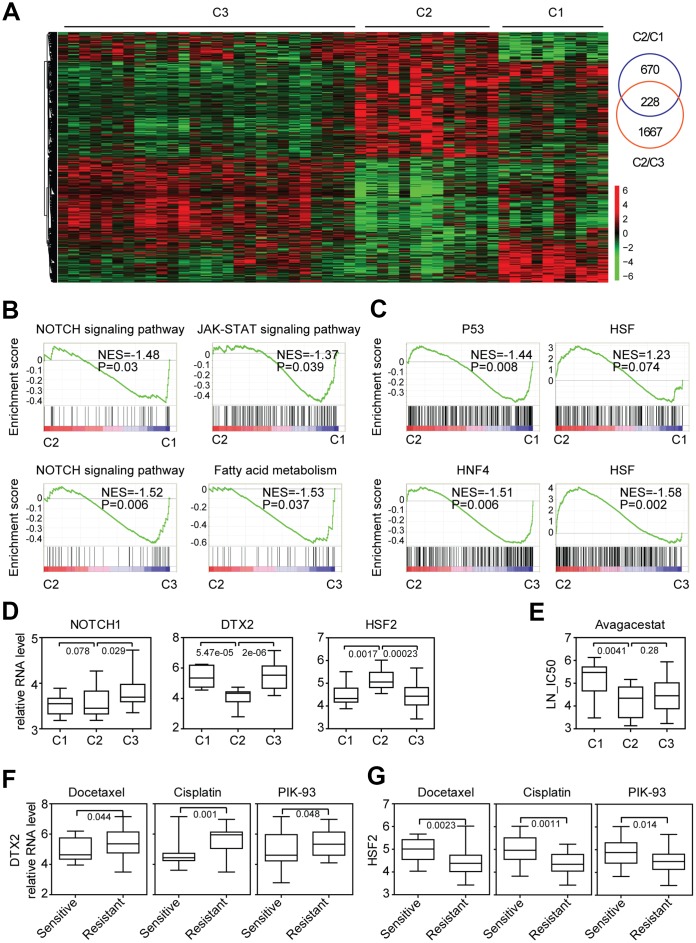
**Characteristics of gene expression and functional annotation of colon cancer sub-clusters suggest the different response to NOTCH inhibitor treatment.** (**A**) Un-supervised clustering heatmap showed the genes specifically expressed in cluster2 colon cancer cells. (**B**) Enrichment plots showed the enriched signaling pathways in cluster2 colon cancer cells. Enrichment of NES and P values were shown. (**C**) Enrichment plots showed the enriched transcription factors in cluster2 colon cancer cells. (**D**) Box plots showed the NOTCH1, DTX2 and HSF2 expression in each sub-cluster of colon cancer cells. (**E**) Box plots showed the LN-IC50 of NOTCH signaling pathway inhibitor avagacestat in each sub-cluster of colon cancer cells. (**F**–**G**) Box plots showed the DTX2 (**F**) and HSF2 (**G**) expression in docetaxel, cisplatin, PIK-93 sensitive and resistant colon cancer cells.

To further gain characteristics of the three sub-clusters of colon cancer cells, we identified the different gene expression of each sub-cluster. We focused on the transcriptional characteristics of cluster2. Compared with cluster1, 898 genes were differently expressed in cluster2. And 1895 genes were differently expressed in cluster2, compared with cluster3. Among them, 228 genes were overlapped and distinguished cluster2 from other two clusters (Figure 4A).

To reveal the transcriptional property of colon cancer cells in cluser2, we identified the enriched signaling pathways using GSEA assay [28]. Compared with cluster1 and cluster3, NOTCH signaling pathway, Fatty acid metabolism signaling pathway and JAK-STAT signaling pathway were negatively associated with cluster2 colon cancer cells (Figure 4B). NOTCH1 and DTX2 were important factors in NOTCH signaling pathway and therapeutic targets in colon cancer treatment [29]. We found that compared with cluster3, the expressions of NOTCH1 and DTX2 were particularly lower in cluster2 colon cancer cells (Figure 4D).

Except signaling pathways, the transcription factors enriched in cluster2 colon cancer cells were also identified. We noticed that transcription factor TP53 was highly enriched in cluster1 and transcription factor HNF4 was highly enriched in cluster3 colon cancer cells (Figure 4C). Interestingly, transcription factor HSF was positively associated with cluster2 colon cancer cells (Figure 4C). And the expression of HSF2 was particularly higher in cluster2 colon cancer cells (Figure 4D).

Since, NOTCH signaling pathway was negatively associated with cluster2 and the expressions of NOTCH1 and DTX2 were particularly lower in cluster2 colon cancer cells, we speculated that the NOTCH inhibitors may have different sensitivity in the three sub-clusters of colon cancer cells. Two NOTCH signaling pathway inhibitors Z-LLNle-CHO and avagacestat were analyzed. Compared with cluster1, colon cancer cells in cluster2 were more sensitive to avagacestat but not Z-LLNle-CHO treatment (Figure 4E).

To further state the importance of DTX2 and HSF2 in determining the sensitivity of the chemotherapy and PI3K-mTOR inhibitors, the colon cancer cells were divided into drug sensitive or resistant subtypes based on the scale of drug LN-IC50. Gene expression profiles associated with the sensitivity of chemotherapy and PI3K-mTOR inhibitors treatment were identified. Consistent with the low expression of DTX2 and high expression of HSF2 in cluster2 (Figure 4D), DTX2 was highly expressed in docetaxel, cisplatin and PIK-93 resistant colon cancer cells (Figure 4F), while, HSF2 was highly expressed in docetaxel, cisplatin and PIK-93 sensitive colon cancer cells (Figure 4G).

### Lack of CDX2 expression is associated with the sensitivity of chemotherapy, BRAF inhibitors and PI3K-mTOR inhibitors

So far, we identified a molecular sub-cluster of colon cancer cells which was sensitive to chemotherapy, BRAF inhibitors, PI3K-mTOR inhibitors and NOTCH inhibitors treatment. Above results suggested that those drugs shared some similar inner mechanisms in determining their sensitivity. And common molecular markers could be used to predict the efficiency of those drugs.

**Figure 5:**
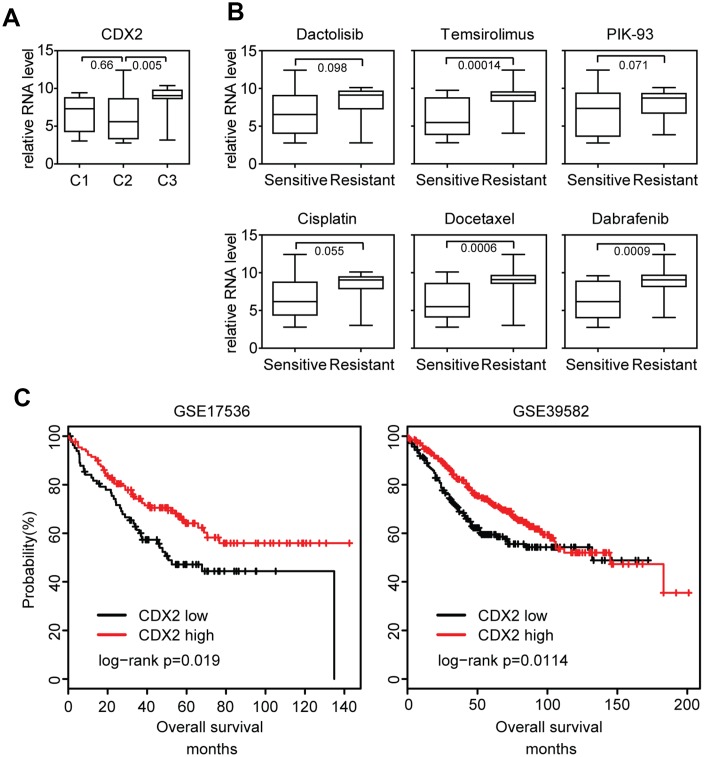
**Lack of CDX2 expression is associated with the sensitivity of chemotherapy, BRAF inhibitors and PI3K-mTOR inhibitors.** (**A**) Box plots showed the CDX2 expression in each sub-cluster of colon cancer cells. (**B**) Box plots showed the CDX2 expression in docetaxel, cisplatin, PIK-93 sensitive and resistant colon cancer cells. (**C**) Relationships of CDX2 expression and overall survival were analyzed from GSE17536 and GSE39582 datasets. Kaplan Meier survival analysis was used to compare the overall survival between CDX2 highly expressed colon cancer patients and CDX2 lowly expressed colon cancer patients. P values were determined by Log-rank test.

CDX2 could suppress intestinal cancer development [30] and is a critical biomarker in colon cancer prognosis [31]. A subgroup of colon cancer patients with lack of CDX2 expression preferentially benefits from adjuvant chemotherapy. We then determined the expression of CDX2 in different colon cancer sub-clusters. Consistent with the observations in colon cancer patients, we found that CDX2 was particularly down regulated in cluster2 colon cancer cells (Figure 5A), which were sensitive to chemotherapy treatment.

Since cluster2 colon cancer cells were also sensitive to BRAF inhibitors and PI3K-mTOR inhibitors treatment, we further identified the association between CDX2 expression and the sensitivity of BRAF inhibitors or PI3K-mTOR inhibitors. We found that CDX2 was highly expressed in docetaxel, cisplatin, temsirolimus, dabrafenib and PIK-93 resistant colon cancer cells (Figure 5B). Those results suggested that the sub-cluster of colon cancer patients with lack of CDX2 expression not only could benefit from adjuvant chemotherapy, but also preferentially benefit from BRAF inhibitors or PI3K-mTOR inhibitors treatment.

We also showed that CDX2 high expression was associated with better prognostic outcomes in GSE17536 [32] and GSE39582 [8] expression datasets (Figure 5C). Based on those results and previous published data, we speculated that genes with similar expression profiling of CDX2 were also important prognostic biomarkers for colon cancer patients.

### Lack of VDR expression is associated with the sensitivity of chemotherapy, BRAF inhibitors and PI3K-mTOR inhibitors

**Figure 6:**
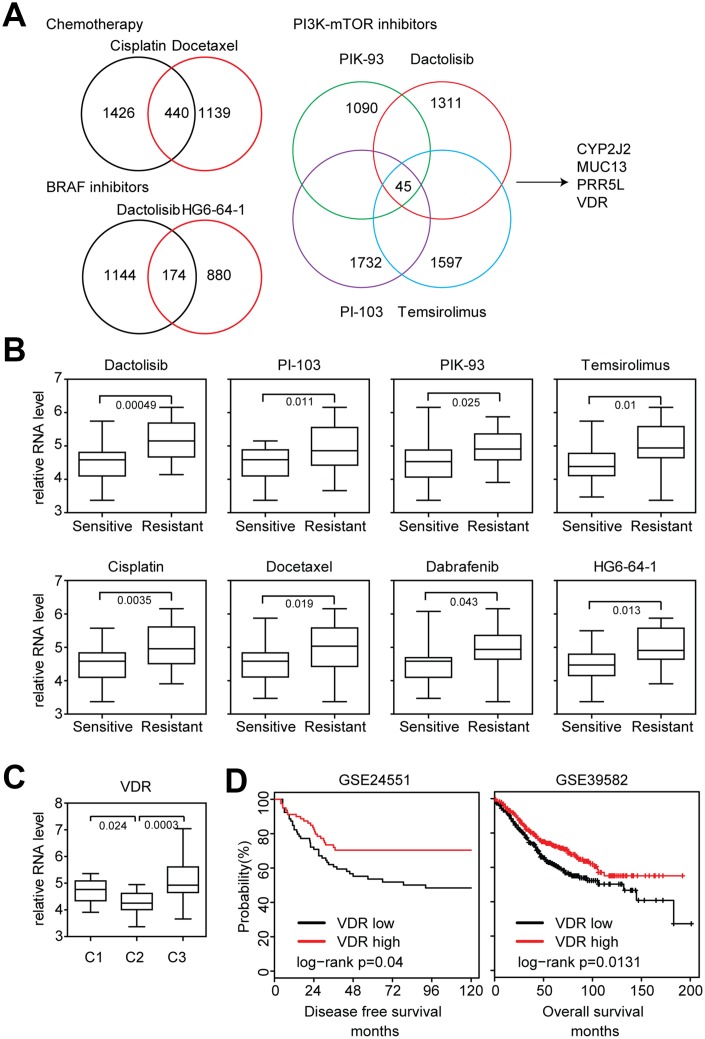
**Lack of VDR expression is associated with the sensitivity of chemotherapy, BRAF inhibitors and PI3K-mTOR inhibitors.** (**A**) Venny diagrams depicted the common genes associated with chemotherapy, BRAF inhibitors and PI3K-mTOR inhibitors sensitivity. (**B**) Box plots showed the VDR expression in chemotherapy, BRAF inhibitors, PI3K-mTOR inhibitors sensitive and resistant colon cancer cells. (**C**) Box plots showed the VDR expression in each sub-cluster of colon cancer cells. (**D**) Relationships of VDR expression and disease free survival or overall survival were analyzed from GSE24551 and GSE39582 datasets.

To identify additional prognostic biomarkers associated with the drug sensitivity in colon cancer cells, different gene expression profiles between sensitive and resistant colon cancer cells responding to the chemotherapy, BRAF inhibitors and PI3K-mTOR inhibitors treatment were identified. 440 genes were differently expressed in cisplatin and docetaxel resistant colon cancer cells (Figure 6A). 178 genes were differently expressed in dactolisib and HG6-64-1 BRAF inhibitors resistant colon cancer cells (Figure 6A). And 45 genes were differently expressed in PI3K-mTOR inhibitors resistant colon cancer cells (Figure 6A). Interestingly, we found four genes CYP2J2, MUC13, PRR5L and VDR were all associated with the sensitivity of chemotherapy, BRAF inhibitors and PI3K-mTOR inhibitors (Figure 6A).

VDR played important roles in intestinal tumorigenesis [33, 34]. However, the association between VDR expression and drug sensitivity in colon cancer cells was not clear. We found that VDR was highly expressed in chemotherapy, BRAF inhibitors and PI3K-mTOR inhibitors resistant colon cancer cells (Figure 6B). VDR was also particularly down regulated in cluster2 colon cancer cells (Figure 6C). And VDR high expression was associated with better prognostic outcomes in colon cancer patients derived from GSE24551 [35] and GSE39582 [8] expression datasets (Figure 6D). All those VDR expression features were quite similar with CDX2, so we speculated that VDR was also an important prognostic biomarker for colon cancer patients. And a sub-cluster of colon cancer patients with lack of VDR expression could benefit from adjuvant chemotherapy, BRAF inhibitors and PI3K-mTOR inhibitors treatment.

### The VDR and CDX2 mediated transcriptional networks

**Figure 7:**
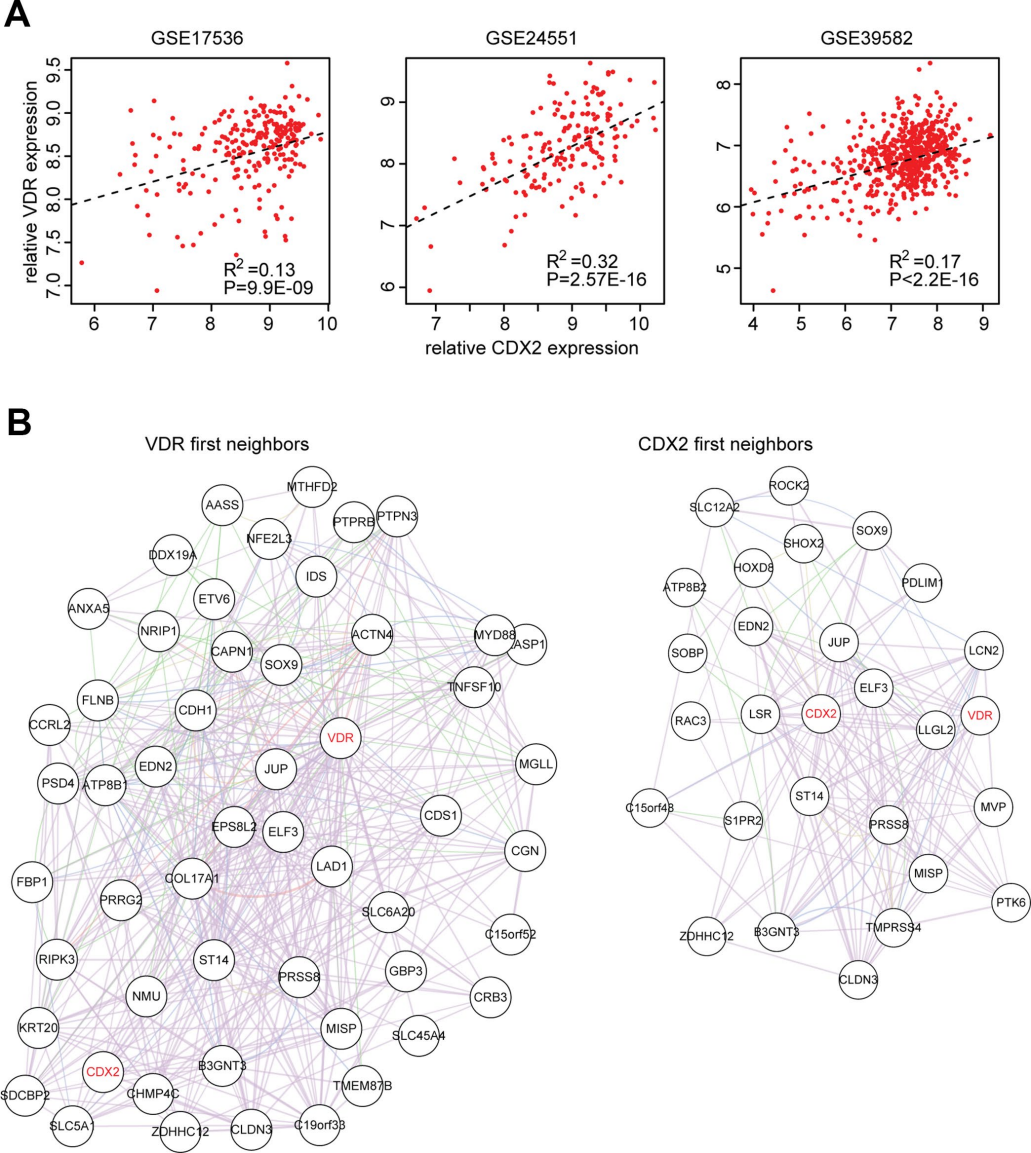
**The VDR and CDX2 mediated transcriptional networks.** (**A**) Spearman correlation between VDR and CDX2 expression in GSE17536, GSE24551 and GSE39582 datasets derived from primary colon cancer patients. (**B**) VDR and CDX2 mediated regulatory gene networks were created by cytoscape. First neighbor genes connected with VDR or CDX2 were demonstrated.

Our results demonstrated the similar functions of VDR and CDX2 in determining the chemotherapy, BRAF inhibitors and PI3K-mTOR inhibitors sensitivity, so, we tried to determine the connection between VDR and CDX2. Spearman correlation demonstrated a positive correlation between VDR and CDX2 expression in three published GEO expression datasets derived from primary colon cancer patients (Figure 7A).

To further explore VDR, CDX2 and their connection to downstream target genes, VDR and CDX2 mediated regulatory networks were constructed using cluster2 specific genes. As expected, VDR was connected with CDX2 from VDR first neighbor genes and CDX2 first neighbor genes (Figure 7B). Functions of VDR and CDX2 associated genes in regulating the sensitivity of chemotherapy, BRAF inhibitors and PI3K-mTOR inhibitors should be further studied.

### Validation of NMF sub-cluster classification in colon cancer clinical patients

Our data showed that when divided into three or four sub-clusters by NMF classification, each cluster of colon cancer cells demonstrated distinctive response to BRAF inhibitors or PI3K-mTOR inhibitors treatment. However, previous results showed five distinctive sub-clusters identified by NMF classification in colon cancer clinical patients [15].

**Figure 8:**
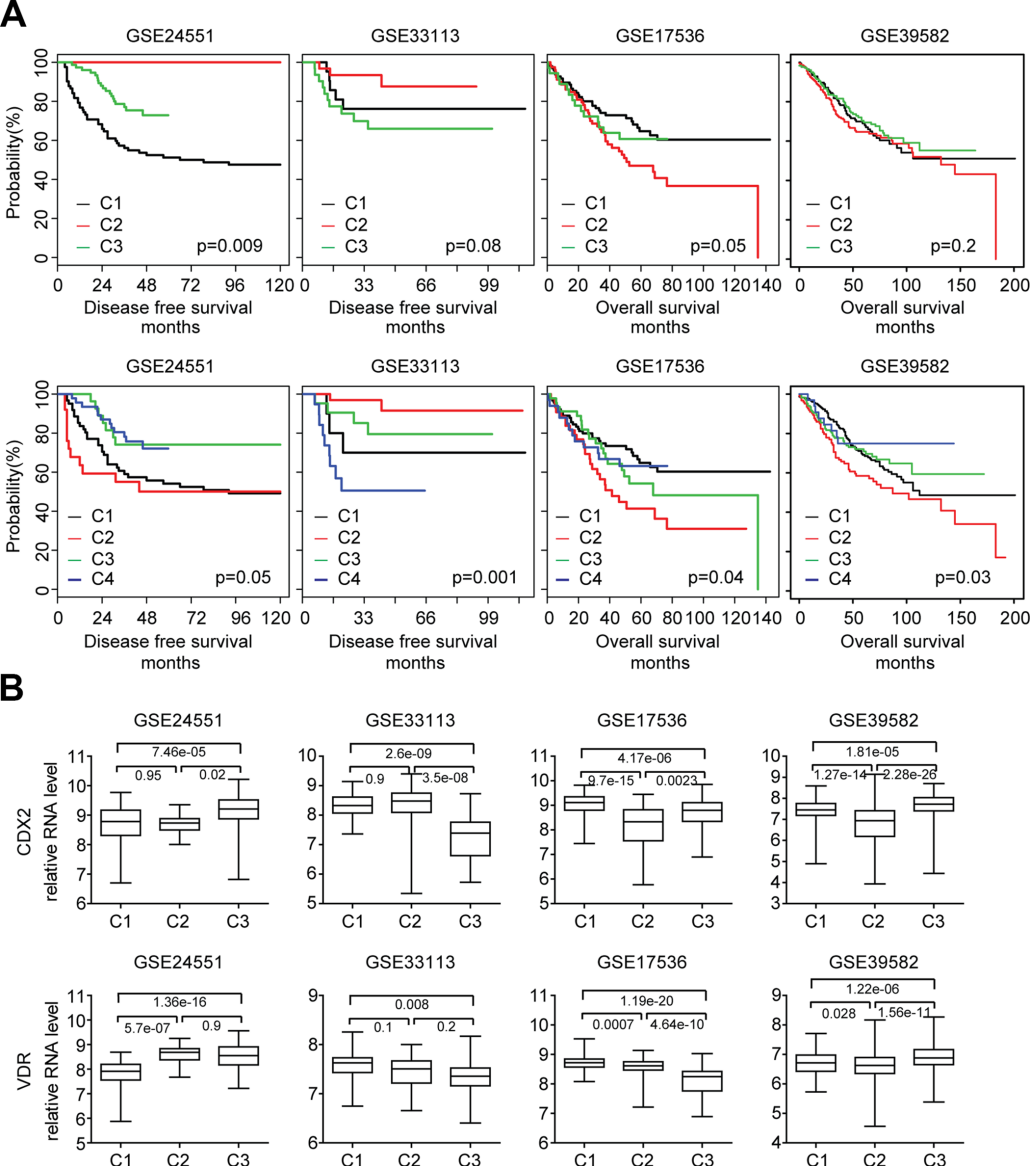
**Validation of NMF sub-cluster classification in colon cancer clinical patients.** (**A**) Primary colon cancer patients from GSE24551, GSE33113, GSE17536 and GSE39582 datasets were divided into three or four sub-clusters based on the gene expression profiling using NMF. Kaplan Meier survival analysis was used to determine the disease free survival or overall survival in three or four sub-clusters of colon cancer patients. (**B**) Box plots showed the CDX2 and VDR expression in each sub-cluster of colon cancer patients.

To address this inconsistency, we used published primary colon cancer expression datasets. Using NMF classification, colon cancer patients from four GEO datasets were divided into the three sub-clusters or four sub-clusters based on the globe transcriptional profiling. Then the disease free survival or overall survival of each sub-cluster of colon cancer patients was determined. When divided into three sub-clusters, there was significant difference in disease free survival or overall survival of each sub-cluster of colon cancer patients in GSE24551 and GSE17536 datasets (Figure 8A). When dividing into four sub-clusters, there was significant difference in disease free survival or overall survival of each sub-cluster of colon cancer patients in GSE24551, GSE33113, GSE17536 and GSE39582 four datasets (Figure 8A). Those results suggested that consistent with the three sub-clusters of colon cancer cell lines, three or four sub-clusters could distinguish colon cancer patients from each other and each sub-cluster was with different clinical outcomes. CDX2 and VDR expression level in the three sub-clusters of colon cancer patients were also significantly different (Figure 8B). Those results further confirmed that CDX2 and VDR were important biomarkers associated with different colon cancer sub-clusters.

Overall, our results showed a molecular sub-cluster of colon cancer cells with low CDX2 and VDR expression was sensitive to chemotherapy, BRAF inhibitors and PI3K-mTOR inhibitors treatment. Detection of CDX2 and VDR expression will provide additional information to ensure the success of chemotherapy, BRAF inhibitor or PI3K-mTOR inhibitor therapy in colon cancer patients.

## DISCUSSION

In this study, we use two colon cancer classification systems CMS and NMF to identify the intrinsic subtypes of colon cancer cell lines based on the gene expression profiling. Because cancer cell lines are devoid the influences of the tumor microenvironment, the classifications of colon cancer cell lines would be more likely to reflect the intrinsic heterogeneity of colon cancer cells. In the CMS classification, CMS1 colon cancer cells are all MSI status (Figure 1C), consistent with the results identified from colon cancer patients. However, there is no significant difference in the frequencies of BRAF, K-Ras and APC mutation in CMS subtypes derived from colon cancer cell lines (Figure 1C). In the NMF classification, we report the discovery of at least three genomic sub-clusters of colon cancer cells with different TP53, BRAF, PIK3CA and POLE mutation frequency (Figure 3A). However, the three sub-clusters of colon cancer cells show no difference in the MSI status distribution (Figure 3A).

The subgroups of colon cancer cell lines derived from CMS and NMF classification systems also demonstrate quite different therapeutic characteristics in responding to anti-cancer drug treatment. CMS3 subtype colon cancer cells are more sensitive to 5-Fluorouracil treatment. And CMS4 subtype colon cancer cells are more sensitive to cisplatin treatment (Figure 1D). In the NMF classification, we find that a molecular sub-cluster of colon cancer cells is specifically sensitive to chemotherapy, BRAF inhibitors, PI3K-mTOR inhibitors and NOTCH inhibitor treatment (Figure 2C, 3B, 3C and 4E). However, EGFR inhibitors have no drug preference in CMS or NMF subtypes (Figure 1E and 2D). We think those two classification systems are quite useful in selection of colon cancer treatment strategies. Since CMS is a robust classification of MSI status in both colon cancer patients and colon cancer cell lines, it may provide significant prognostic information to evaluate colon cancer patients who are selected to receive immune checkpoint therapeutic treatment [36, 37]. However, for NMF classification, it may provide more prognostic information to predict the clinical response to chemotherapy, BRAF inhibitors, PI3K-mTOR inhibitors and NOTCH inhibitor.

Targets of chemotherapy, BRAF inhibitors and PI3K-mTOR inhibitors are quite different. However, a molecular sub-cluster of colon cancer cells is specifically sensitive to chemotherapy, BRAF inhibitors and PI3K-mTOR inhibitors treatment suggesting those drugs share some similar inner mechanisms to determine their sensitivity. And common molecular markers could be used to predict the efficiency of those drugs. CDX2 is used to predict the benefit of adjuvant chemotherapy in colon cancer patients. Additionally, our results suggest that CDX2 expression is also associated with the sensitivity of BRAF inhibitors and PI3K-mTOR inhibitors treatment (Figure 5B).

VDR is a new identified molecular marker to predict the efficiency of those drugs. The VDR expression features are quite similar with CDX2. For example, both VDR and CDX2 are highly expressed in chemotherapy, BRAF inhibitors and PI3K-mTOR inhibitors resistant colon cancer cells (Figure 5B and 6B). Both VDR and CDX2 are lowly expressed in cluster2 subtypes of colon cancer cells (Figure 5A and 6C). Both VDR and CDX2 are associated with better clinical outcomes (Figure 5C and 6D). Moreover, there is a positive correlation between VDR and CDX2 expression in primary colon cancer patients (Figure 7A and 7B). All those results suggest that similar with CDX2, VDR is also an important prognostic biomarker for colon cancer patients. And the sub-cluster of colon cancer patients with lack of VDR expression could benefit from adjuvant chemotherapy, BRAF inhibitors and PI3K-mTOR inhibitors treatment. However, those results should further be tested in clinical colon cancer patients.

Overall, our results demonstrate a molecular sub-cluster of colon cancer cells with low CDX2 and VDR expression is sensitive to chemotherapy, BRAF inhibitors and PI3K-mTOR inhibitors treatment and provide an example of translation of colon cancer classification to subgroups guided targeted therapies.

## MATERIALS AND METHODS

### Data collection

Gene expression, genomic mutation and drug sensitivity of colon cancer cell lines were downloaded from Genomics of Drug Sensitivity in Cancer project (https://www.cancerrxgene.org/). Gene expression series matrix of colon cancer patients with clinical disease free survival or overall survival were downloaded from GEO website (https://www.ncbi.nlm.nih.gov/geo/) with GEO number GSE24551, GSE33113, GSE17536 and GSE39582.

### Gene expression data processing

The matrix file of each GEO dataset was annotated by corresponding platform. The expression values were averaged if multiple probes corresponded to the same gene symbol using R software “plyr” package. The “plyr” package and the basic usage were downloaded from bioconductor (https://cran.r-project.org/web/ packages/plyr/index.html).

### The consensus molecular subtypes (CMS) classification of colon cancer cell lines

Colon cancer cell lines were divided into CMS1, CMS2, CMS3 and CMS4 subtypes by “CMScaller”. “CMScaller” is available as an R package and could be downloaded from bioconductor. The basic usage of “CMScaller” was described in [21].

### The Nonnegative Matrix Factorization (NMF) classification of colon cancer cell lines and colon cancer patients

Colon cancer cell lines or colon cancer patients were divided into two sub-clusters, three sub-clusters or four sub-clusters by R software “NMF” package by rank=2, rank=3 or rank=4. The “NMF” package and the basic usage were downloaded from bioconductor (https://cran.r-project.org/web/packages/NMF/index.html).

### Heatmap presentation

Heatmaps were created by “pheatmap” package. “pheatmap” is available as an R package and could be downloaded from bioconductor (https://cran.r-project.org/web/packages/pheatmap/index.html). The clustering scale was determined by “average” method.

### Venny diagram

The venny diagrams were generated by VENNY 2.1 (http://bioinfogp.cnb.csic.es/tools/venny/index.html).

### Gene set enrichment analysis (GSEA)

Gene set enrichment analysis was performed using GSEA 2.0. The GSEA software and gene sets were downloaded from the GSEA Web site (http://www.broad.mit.edu/gsea/index.html). Genes ranked by signal-to-noise ratio, and statistical significance was determined by 1,000 gene set permutations. Gene set enriched signaling pathways and transcription factors were identified.

### Identification of genes associated with the drug sensitivity

Colon cancer cell lines were classified into drug sensitive or resistant sub-groups based on the scale values of the LN-IC50 using the “scale” method of R software. Different gene expression between drug sensitive or resistant colon cancer cells was identified by Student’s t test.

### Survival analysis

Kaplan-Meier estimator was applied to identify the influence of CDX2 or VDR expression on overall survival or disease free survival using “survival” package in the R statistics software. The overall survival or disease free survival of each colon cancer sub-cluster was also determined by “survival” package. The “survival” package and the basic usage were downloaded from bioconductor (https://cran.r-project.org/web/packages/survival/index.html). P values were determined by Log-rank test.

### Spearman correlation

Spearman correlation was used to study the correlation between CDX2 expression and VDR expression in colon cancer patients using the “lm” method of R software.

### CDX2 or VDR associated transcriptional network

The networks of cluter2 specific genes were created by Cytoscape GeneMANIA App. The first degrees of CDX2 or VDR connected genes were demonstrated.

### Statistical analysis

The box plots and contingency graphs were generated from prims5.0. Statistical analysis was performed using the Student’s t test or Chi-square test. P value less than 0.05 was chosen to be statistically significant difference unless specifically notified.

## References

[1] Sottoriva A, Kang H, Ma Z, Graham TA, Salomon MP, Zhao J, Marjoram P, Siegmund K, Press MF, Shibata D, Curtis C. A Big Bang model of human colorectal tumor growth. Nat Genet. 2015; 47:209–16. 10.1038/ng.3214 25665006 PMC4575589

[2] Sveen A, Løes IM, Alagaratnam S, Nilsen G, Høland M, Lingjærde OC, Sorbye H, Berg KC, Horn A, Angelsen JH, Knappskog S, Lønning PE, Lothe RA. Intra-patient Inter-metastatic Genetic Heterogeneity in Colorectal Cancer as a Key Determinant of Survival after Curative Liver Resection. PLoS Genet. 2016; 12:e1006225. 10.1371/journal.pgen.1006225 27472274 PMC4966938

[3] Dienstmann R, Vermeulen L, Guinney J, Kopetz S, Tejpar S, Tabernero J. Consensus molecular subtypes and the evolution of precision medicine in colorectal cancer. Nat Rev Cancer. 2017; 17:79–92. 10.1038/nrc.2016.126 28050011

[4] Bramsen JB, Rasmussen MH, Ongen H, Mattesen TB, Ørntoft MW, Árnadóttir SS, Sandoval J, Laguna T, Vang S, Øster B, Lamy P, Madsen MR, Laurberg S, et al. Molecular-Subtype-Specific Biomarkers Improve Prediction of Prognosis in Colorectal Cancer. Cell Rep. 2017; 19:1268–80. 10.1016/j.celrep.2017.04.045 28494874

[5] Linnekamp JF, Wang X, Medema JP, Vermeulen L. Colorectal cancer heterogeneity and targeted therapy: a case for molecular disease subtypes. Cancer Res. 2015; 75:245–49. 10.1158/0008-5472.CAN-14-2240 25593032

[6] Liu Y, Sethi NS, Hinoue T, Schneider BG, Cherniack AD, Sanchez-Vega F, Seoane JA, Farshidfar F, Bowlby R, Islam M, Kim J, Chatila W, Akbani R, et al. Comparative Molecular Analysis of Gastrointestinal Adenocarcinomas. Cancer Cell. 2018; 33:721–735 e728. 10.1016/j.ccell.2018.03.010 29622466 PMC5966039

[7] Sinicrope FA, Shi Q, Smyrk TC, Thibodeau SN, Dienstmann R, Guinney J, Bot BM, Tejpar S, Delorenzi M, Goldberg RM, Mahoney M, Sargent DJ, Alberts SR. Molecular markers identify subtypes of stage III colon cancer associated with patient outcomes. Gastroenterology. 2015; 148:88–99. 10.1053/j.gastro.2014.09.041 25305506 PMC4274188

[8] Marisa L, de Reyniès A, Duval A, Selves J, Gaub MP, Vescovo L, Etienne-Grimaldi MC, Schiappa R, Guenot D, Ayadi M, Kirzin S, Chazal M, Fléjou JF, et al. Gene expression classification of colon cancer into molecular subtypes: characterization, validation, and prognostic value. PLoS Med. 2013; 10:e1001453. 10.1371/journal.pmed.1001453 23700391 PMC3660251

[9] Fu T, Pappou EP, Guzzetta AA, Jeschke J, Kwak R, Dave P, Hooker CM, Morgan R, Baylin SB, Iacobuzio-Donahue CA, Wolfgang CL, Ahuja N. CpG island methylator phenotype-positive tumors in the absence of MLH1 methylation constitute a distinct subset of duodenal adenocarcinomas and are associated with poor prognosis. Clin Cancer Res. 2012; 18:4743–52. 10.1158/1078-0432.CCR-12-0707 22825585 PMC3482463

[10] Zhang B, Wang J, Wang X, Zhu J, Liu Q, Shi Z, Chambers MC, Zimmerman LJ, Shaddox KF, Kim S, Davies SR, Wang S, Wang P, et al, and NCI CPTAC. Proteogenomic characterization of human colon and rectal cancer. Nature. 2014; 513:382–87. 10.1038/nature13438 25043054 PMC4249766

[11] Vasaikar S, Huang C, Wang X, Petyuk VA, Savage SR, Wen B, Dou Y, Zhang Y, Shi Z, Arshad OA, Gritsenko MA, Zimmerman LJ, McDermott JE, et al. Proteogenomic Analysis of Human Colon Cancer Reveals New Therapeutic Opportunities. Cell. 2019; 177:1035–1049 e1019. 10.1016/j.cell.2019.03.030 31031003 PMC6768830

[12] Guinney J, Dienstmann R, Wang X, de Reyniès A, Schlicker A, Soneson C, Marisa L, Roepman P, Nyamundanda G, Angelino P, Bot BM, Morris JS, Simon IM, et al. The consensus molecular subtypes of colorectal cancer. Nat Med. 2015; 21:1350–56. 10.1038/nm.3967 26457759 PMC4636487

[13] Kwon Y, Park M, Jang M, Yun S, Kim WK, Kim S, Paik S, Lee HJ, Hong S, Kim TI, Min B, Kim H. Prognosis of stage III colorectal carcinomas with FOLFOX adjuvant chemotherapy can be predicted by molecular subtype. Oncotarget. 2017; 8:39367–81. 10.18632/oncotarget.17023 28455965 PMC5503619

[14] Sveen A, Bruun J, Eide PW, Eilertsen IA, Ramirez L, Murumägi A, Arjama M, Danielsen SA, Kryeziu K, Elez E, Tabernero J, Guinney J, Palmer HG, et al. Colorectal Cancer Consensus Molecular Subtypes Translated to Preclinical Models Uncover Potentially Targetable Cancer Cell Dependencies. Clin Cancer Res. 2018; 24:794–806. 10.1158/1078-0432.CCR-17-1234 29242316

[15] Sadanandam A, Lyssiotis CA, Homicsko K, Collisson EA, Gibb WJ, Wullschleger S, Ostos LC, Lannon WA, Grotzinger C, Del Rio M, Lhermitte B, Olshen AB, Wiedenmann B, et al. A colorectal cancer classification system that associates cellular phenotype and responses to therapy. Nat Med. 2013; 19:619–25. 10.1038/nm.3175 23584089 PMC3774607

[16] Tan IB, Ivanova T, Lim KH, Ong CW, Deng N, Lee J, Tan SH, Wu J, Lee MH, Ooi CH, Rha SY, Wong WK, Boussioutas A, et al. Intrinsic subtypes of gastric cancer, based on gene expression pattern, predict survival and respond differently to chemotherapy. Gastroenterology. 2011; 141:476–485, 485 e471–411. 10.1053/j.gastro.2011.04.042 21684283 PMC3152688

[17] Lei Z, Tan IB, Das K, Deng N, Zouridis H, Pattison S, Chua C, Feng Z, Guan YK, Ooi CH, Ivanova T, Zhang S, Lee M, et al. Identification of molecular subtypes of gastric cancer with different responses to PI3-kinase inhibitors and 5-fluorouracil. Gastroenterology. 2013; 145:554–65. 10.1053/j.gastro.2013.05.010 23684942

[18] Barretina J, Caponigro G, Stransky N, Venkatesan K, Margolin AA, Kim S, Wilson CJ, Lehár J, Kryukov GV, Sonkin D, Reddy A, Liu M, Murray L, et al. The Cancer Cell Line Encyclopedia enables predictive modelling of anticancer drug sensitivity. Nature. 2012; 483:603–07. 10.1038/nature11003 22460905 PMC3320027

[19] Ghandi M, Huang FW, Jané-Valbuena J, Kryukov GV, Lo CC, McDonald ER 3rd, Barretina J, Gelfand ET, Bielski CM, Li H, Hu K, Andreev-Drakhlin AY, Kim J, et al. Next-generation characterization of the Cancer Cell Line Encyclopedia. Nature. 2019; 569:503–08. 10.1038/s41586-019-1186-3 31068700 PMC6697103

[20] Iorio F, Knijnenburg TA, Vis DJ, Bignell GR, Menden MP, Schubert M, Aben N, Gonçalves E, Barthorpe S, Lightfoot H, Cokelaer T, Greninger P, van Dyk E, et al. A Landscape of Pharmacogenomic Interactions in Cancer. Cell. 2016; 166:740–54. 10.1016/j.cell.2016.06.017 27397505 PMC4967469

[21] Eide PW, Bruun J, Lothe RA, Sveen A. CMScaller: an R package for consensus molecular subtyping of colorectal cancer pre-clinical models. Sci Rep. 2017; 7:16618. 10.1038/s41598-017-16747-x 29192179 PMC5709354

[22] Cremolini C, Schirripa M, Antoniotti C, Moretto R, Salvatore L, Masi G, Falcone A, Loupakis F. First-line chemotherapy for mCRC—a review and evidence-based algorithm. Nat Rev Clin Oncol. 2015; 12:607–19. 10.1038/nrclinonc.2015.129 26215044

[23] Raoul JL, Van Laethem JL, Peeters M, Brezault C, Husseini F, Cals L, Nippgen J, Loos AH, Rougier P. Cetuximab in combination with irinotecan/5-fluorouracil/folinic acid (FOLFIRI) in the initial treatment of metastatic colorectal cancer: a multicentre two-part phase I/II study. BMC Cancer. 2009; 9:112. 10.1186/1471-2407-9-112 19366444 PMC2678147

[24] Van Cutsem E, Köhne CH, Hitre E, Zaluski J, Chang Chien CR, Makhson A, D’Haens G, Pintér T, Lim R, Bodoky G, Roh JK, Folprecht G, Ruff P, et al. Cetuximab and chemotherapy as initial treatment for metastatic colorectal cancer. N Engl J Med. 2009; 360:1408–17. 10.1056/NEJMoa0805019 19339720

[25] Tol J, Koopman M, Cats A, Rodenburg CJ, Creemers GJ, Schrama JG, Erdkamp FL, Vos AH, van Groeningen CJ, Sinnige HA, Richel DJ, Voest EE, Dijkstra JR, et al. Chemotherapy, bevacizumab, and cetuximab in metastatic colorectal cancer. N Engl J Med. 2009; 360:563–72. 10.1056/NEJMoa0808268 19196673

[26] Mirza A, Wu Q, Wang L, McClanahan T, Bishop WR, Gheyas F, Ding W, Hutchins B, Hockenberry T, Kirschmeier P, Greene JR, Liu S. Global transcriptional program of p53 target genes during the process of apoptosis and cell cycle progression. Oncogene. 2003; 22:3645–54. 10.1038/sj.onc.1206477 12789273

[27] di Blasio L, Puliafito A, Gagliardi PA, Comunanza V, Somale D, Chiaverina G, Bussolino F, Primo L. PI3K/mTOR inhibition promotes the regression of experimental vascular malformations driven by PIK3CA-activating mutations. Cell Death Dis. 2018; 9:45. 10.1038/s41419-017-0064-x 29352118 PMC5833448

[28] Subramanian A, Tamayo P, Mootha VK, Mukherjee S, Ebert BL, Gillette MA, Paulovich A, Pomeroy SL, Golub TR, Lander ES, Mesirov JP. Gene set enrichment analysis: a knowledge-based approach for interpreting genome-wide expression profiles. Proc Natl Acad Sci USA. 2005; 102:15545–50. 10.1073/pnas.0506580102 16199517 PMC1239896

[29] Schmidt EM, Lamprecht S, Blaj C, Schaaf C, Krebs S, Blum H, Hermeking H, Jung A, Kirchner T, Horst D. Targeting tumor cell plasticity by combined inhibition of NOTCH and MAPK signaling in colon cancer. J Exp Med. 2018; 215:1693–708. 10.1084/jem.20171455 29769248 PMC5987917

[30] Balbinot C, Armant O, Elarouci N, Marisa L, Martin E, De Clara E, Onea A, Deschamps J, Beck F, Freund JN, Duluc I. The Cdx2 homeobox gene suppresses intestinal tumorigenesis through non-cell-autonomous mechanisms. J Exp Med. 2018; 215:911–26. 10.1084/jem.20170934 29439001 PMC5839756

[31] Dalerba P, Sahoo D, Paik S, Guo X, Yothers G, Song N, Wilcox-Fogel N, Forgó E, Rajendran PS, Miranda SP, Hisamori S, Hutchison J, Kalisky T, et al. CDX2 as a Prognostic Biomarker in Stage II and Stage III Colon Cancer. N Engl J Med. 2016; 374:211–22. 10.1056/NEJMoa1506597 26789870 PMC4784450

[32] Smith JJ, Deane NG, Wu F, Merchant NB, Zhang B, Jiang A, Lu P, Johnson JC, Schmidt C, Bailey CE, Eschrich S, Kis C, Levy S, et al. Experimentally derived metastasis gene expression profile predicts recurrence and death in patients with colon cancer. Gastroenterology. 2010; 138:958–68. 10.1053/j.gastro.2009.11.005 19914252 PMC3388775

[33] Ferrer-Mayorga G, Gómez-López G, Barbáchano A, Fernández-Barral A, Peña C, Pisano DG, Cantero R, Rojo F, Muñoz A, Larriba MJ. Vitamin D receptor expression and associated gene signature in tumour stromal fibroblasts predict clinical outcome in colorectal cancer. Gut. 2017; 66:1449–62. 10.1136/gutjnl-2015-310977 27053631 PMC5530491

[34] Bhatia V, Falzon M. Restoration of the anti-proliferative and anti-migratory effects of 1,25-dihydroxyvitamin D by silibinin in vitamin D-resistant colon cancer cells. Cancer Lett. 2015; 362:199–207. 10.1016/j.canlet.2015.03.042 25846868 PMC4419377

[35] Agesen TH, Sveen A, Merok MA, Lind GE, Nesbakken A, Skotheim RI, Lothe RA. ColoGuideEx: a robust gene classifier specific for stage II colorectal cancer prognosis. Gut. 2012; 61:1560–67. 10.1136/gutjnl-2011-301179 22213796

[36] Diaz LA Jr, Le DT. PD-1 Blockade in Tumors with Mismatch-Repair Deficiency. N Engl J Med. 2015; 373:1979. 10.1056/NEJMc1510353 26559582

[37] Le DT, Durham JN, Smith KN, Wang H, Bartlett BR, Aulakh LK, Lu S, Kemberling H, Wilt C, Luber BS, Wong F, Azad NS, Rucki AA, et al. Mismatch repair deficiency predicts response of solid tumors to PD-1 blockade. Science. 2017; 357:409–13. 10.1126/science.aan6733 28596308 PMC5576142

